# Conclusions

**Published:** 2008

**Authors:** Robert Hitzemann, Denesa Oberbeck

Although alcohol affects almost all tissues and organs in the body, the brain can be considered the most significant target of alcohol use and/or abuse. Thus, alcohol’s effects on the brain are immediate and widespread, ranging from effects on normal physiology, metabolism, and gait to changes in emotions, cognition, and other functions. Moreover, alcohol’s impact on brain functioning plays a central role in the additive and permanent adaptations, such as the development of alcohol dependence with its features of tolerance or withdrawal upon discontinuation of alcohol use. Therefore, it is crucial to explore alcohol’s effects on the brain at many levels, from cellular and molecular biology studies or isolated cells or cell components to functional imaging studies investigating brain function in the living organism.

To achieve this, an ever-increasing spectrum of techniques and methods has become available in recent years throughout the field of neuroscience. This Special Section has provided a representative sampling of the latest strategies being used by scientists to understand the neural mechanisms of the alcoholic brain. The results obtained using these strategies will not only elucidate the mechanisms through which alcohol acts on specific brain cells or signaling systems but also will allow researchers from a variety of areas to design novel diagnostic approaches as well as develop new intervention strategies (e.g., pharmacotherapies) to treat alcohol dependence and thus reduce the heavy burden the disease places on the individual drinker, his or her family, and society as a whole.

